# Natural polysaccharides as multifunctional anti-cancer agents: structure-activity relationships, mechanisms of action, and therapeutic potential

**DOI:** 10.3389/fimmu.2026.1784957

**Published:** 2026-04-02

**Authors:** Jiaxin Jiang, Xiwu Zhang, Di Han, Qichao Liang, Le Yang, Ling Kong, Yu Guan, Hui Sun, Chang Liu, Ye Sun, Ying Han, Jie Zhang, Xijun Wang

**Affiliations:** 1 State key laboratory of Integration and Innovation of Classic Formula and Modern Chinese Medicine, National Chinmedomics Research Center, National TCM Key Laboratory of Serum Pharmacochemistry, Metabolomics Laboratory, Department of Pharmaceutical Analysis, Heilongjiang University of Chinese Medicine, Harbin, China; 2 State Key Laboratory of Dampness Syndrome, The Second Affiliated Hospital Guangzhou University of Chinese Medicine, Guangzhou, China; 3 Technology Innovation Center of Wusulijiang Ciwujia, Hulin, China

**Keywords:** clinical translation, polysaccharides, selective toxicity, signaling pathways, underlying mechanisms

## Abstract

Cancer remains a formidable global health challenge, characterized by alarmingly high incidence and mortality rates. Traditional clinical therapies are often accompanied by obvious toxicity and side effects, highlighting the urgent need to develop safer and more effective therapeutic alternatives. In recent years, polysaccharides have emerged as promising candidates for anti-tumor drugs due to their wide sources, high biocompatibility and low toxicity. This review summarizes recent advances in anti-tumor effects of polysaccharides, covering their underlying mechanisms, key signaling pathways and selective toxicity characteristics. Polysaccharides exert synergistic anti-cancer effects through multi-target, multi-pathway mechanisms, including the induction of immune cell polarization and tumor cell apoptosis, inhibition of tumor cell migration and angiogenesis, and modulation of key signaling pathways such as P53, NF-κB, and Wnt/β-catenin. Among these, polysaccharides with specific monosaccharide compositions, optimal molecular weights, β-glycosidic linkages, triple-helix conformations, or those that are chemically modified, exhibit enhanced biological and anti-tumor activities. Future efforts should focus on elucidating structure-activity relationships, developing targeted delivery systems to improve bioavailability and tumor specificity, and advancing large-scale, multi-center, long-term clinical trials to support the development of safe and effective polysaccharide-based anti-cancer therapeutics.

## 1 Introduction

Cancer is a major global health problem as it has the highest incidence and mortality rates (1, 2). There are several available options for treatment, including surgery, radiotherapy, chemotherapy, targeted therapy, and immunotherapy (3). These treatment options do not fully remove the tumors and negatively affect the quality of life for the patients (4). Therefore, finding high-efficacy, low-toxicity anti-cancer drugs along the lines of the existing treatment options is a valued pursuit.

**Figure 1:**
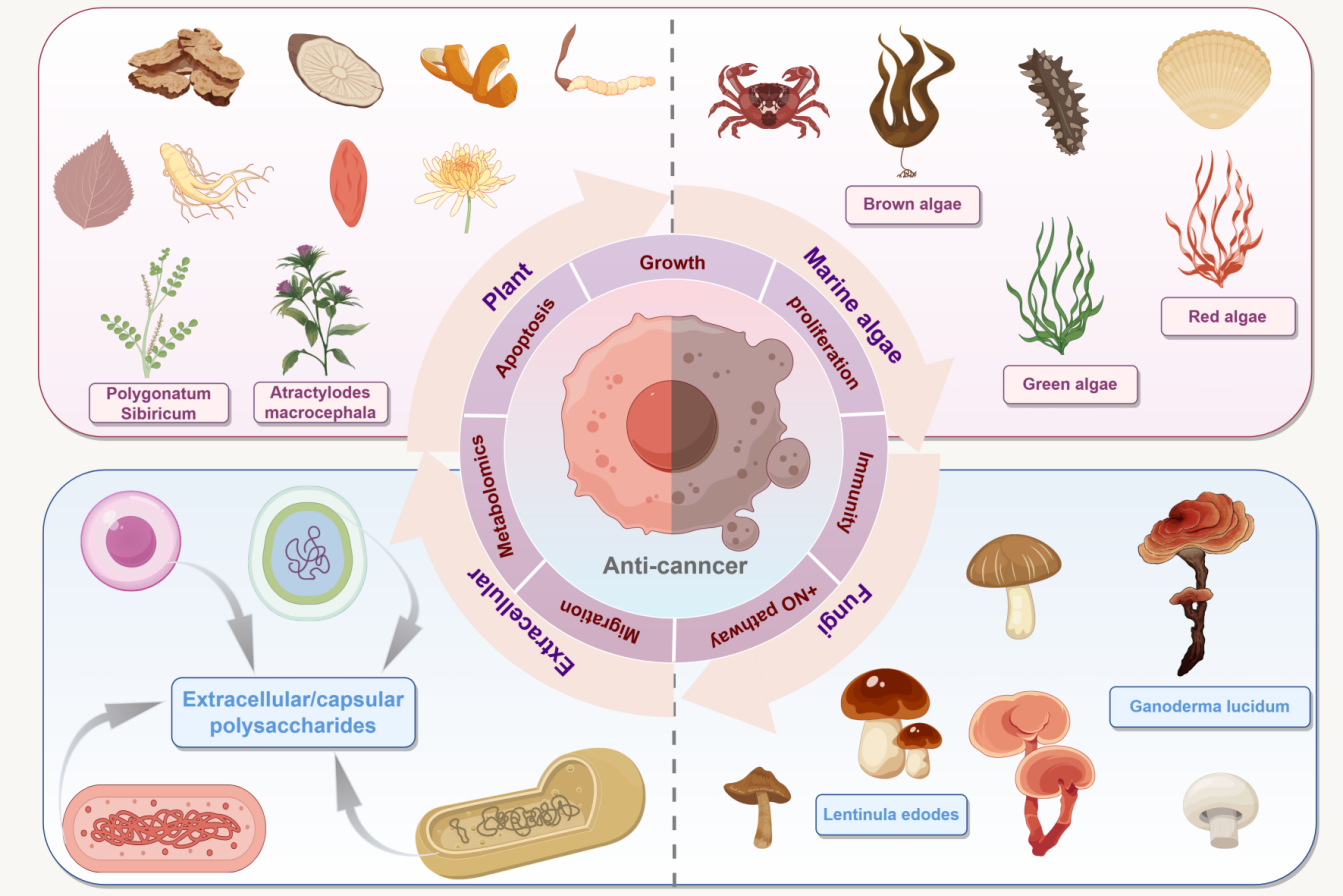
Sources of polysaccharides. Polysaccharides derived from plants, fungi, marine algae, and microorganisms exert anticancer effects through six primary mechanisms: growth inhibition, apoptosis induction, immunomodulation, NO pathway activation, metastasis suppression, and metabolic regulation, et al. (Draw using figshare: https://www.figdraw.com/. The authorized ID: IRAPI09f3f).

Polysaccharides, have attracted growing interest due to their wide availability, low cost, and high safety profile (5, 6). These high molecular weight (MW) compounds are composed of multiple saccharide units joined together by glycosidic bonds, and are present in plants, fungi, algae, and microorganisms (7) (**Figure 1**). As unique natural products, polysaccharides offer several advantages that surpass those of traditional therapeutic drugs. While numerous natural products-including flavonoids, alkaloids, terpenoids, and quinones-exhibit anti-tumor activity, polysaccharides are distinguished by their unique physicochemical properties and multifaceted biological actions. Crucially, unlike cytotoxic small-molecule drugs that indiscriminately target proliferating cells, polysaccharides operate primarily through immunomodulation-engaging multiple immune checkpoints and effector pathways-while concurrently exerting selective cytotoxicity against malignant cells and sparing normal tissues. This dual functionality-potent anti-tumor efficacy coupled with low systemic toxicity and exceptional biocompatibility-directly addresses the dose-limiting toxicity that severely constrains the clinical utility of traditional chemotherapy. Bibliometric analysis of the past two decades reveals a marked increase in publications centered on keywords such as “polysaccharides,” “anti-cancer mechanism,” and “chemotherapeutics,” underscoring escalating scientific interest and the need for a comprehensive synthesis of accumulated evidence. Despite this wealth of data, a cohesive, evidence-based synthesis integrating mechanistic insights, structure-activity relationships, natural sources, selectivity profiles, and translational progress remain absent. This review addresses this critical gap by critically synthesizing current preclinical and clinical evidence, emphasizing how structural features-molecular weight, glycosidic linkages, monosaccharide composition, spatial conformation, chemical modifications-dictate biological function and therapeutic efficacy. By systematically evaluating the cumulative evidence, this work provides a rigorous foundation to guide rational drug development and accelerate clinical translation. Importantly, although other natural and synthetic compounds demonstrate anti-cancer potential, polysaccharides hold exceptional promise-not merely as adjunctive agents, but as viable alternatives to conventional therapies-owing to their tumor-selective action, favorable safety margin, and capacity to restore endogenous anti-tumor immunity.

This review aims to advance the understanding of polysaccharides’ anti-tumor action, their metabolic pathways, their clinical applications, and the current limitations hindering translation from theory to practice. This serves as a foundation for developing viable polysaccharide-based anti-cancer treatments.

## 2 The therapeutic mechanism of polysaccharides on cancer

(**Table 1**)Polysaccharides exert anti-tumor effects through multiple interrelated mechanisms, including immune regulation, inhibition of proliferation, induction of apoptosis, activation of the nitric oxide (NO) pathway, and suppression of migration and angiogenesis. These mechanisms collectively contribute to their extensive anti-cancer activity.

**Table 1:** Polysaccharides regulate immune cells to anti-cancer.

Mechanism	Polysaccharides	Regulatory pathway	Activity immunity	Ref
Regulation of Immune Cells	Pueraria root polysaccharides	Macrophages (RAW 264.7, M2 to M1 phenotype), dendritic cells, natural killer cells, T cell, B cell.	T-cytotoxic cells and B-cell NK, IL-4, IFN-y and TNF-γ↑; macrophage proliferation, NO↑	(8–10)
	*Polygonatum Sibiricum* polysaccharide (PSP)	Macrophages	CD86^+^, M1 phenotype↑; CD206^+^, M2 phenotype↓	(11)
	Radix Bupleuri polysaccharide	T cell	CD4^+^ T cell, MAPK and NF-κB signaling pathways↑	(12)
	Bacterial exopolysaccharides	T cell, natural killer cells, dendritic cells	Th1 T cells, IL-12, TNF-α↑	(13)
	Fungal polysaccharides	Macrophages, T cell	M1 polarization, CD40, CD80, CD86, MHC-II, CD44^+^, CD62L^+^, TCF1^+^↑; TIM-3 and CD317↓	(14, 15)
	Ginsenoside polysaccharides	T cell, B cell.	Thymus and spleen weight, IL-10, TNF-α, IL-6, PI3K/AKT signaling pathway↑	(16)
	Pectic polysaccharide PEP-1	Macrophages	M1 phenotype, phosphorylation of NF-κB and MAPK↑; M2 phenotype↓	(17)
Inhibition of Cancer Cell Proliferation	Phosphorylated fucoidan-natural product, Sulfated galactan	G2/M, G0/G1 phase arrest and apoptosis	Cyclin-dependent kinase inhibitor p21, p53↑; epithelial-mesenchymal transition, cyclin-D, cyclin-E, cdk-4, cdk-2, EGFR↓	(18–20)
	*Hedyotis diffusa* polysaccharide	G0/G1 phase arrest	Caspase-3, -8, and -9↑; Bcl-2↓	(21)
	Safflower polysaccharide	G0/G1 phase arrest	Bax, cleaved caspase-3↑; Bcl-2, COX-2↓	(22)
Promotion of Cancer Cell Apoptosis	Algal polysaccharides (Homogeneous sulfated polysaccharide SHA1P-2, sulfated alginate polysaccharide TGC161, fucoidan, etc.)	T cell proliferation, promotes the apoptosis of tumor-associated macrophages,	Phosphorylated IRF3, Caspase-3, PARP, TNF↑; STING-TBK1-IRF3 signaling pathway, phosphorylated IRF3, IL-1β, IL-6, and TNF-α↓	(23–32)
	Hedyotis diffusa polysaccharide	G0/G1 phase arrest	Caspase 3, caspase 8, caspase 9↑; Bcl-2↓	(21)
	Pleurotus ostreatus polysaccharide	G0/G1 phase arrest	Caspase-9 and Bax proteins↑; P53, cyclin D, and Cdk4↓	(33)
	Safflower polysaccharide	G0/G1 phase arrest	Bax and cleaved caspase-3↑; Bcl-2 and COX-2↓	(20)
	Peach gum polysaccharide	Macrophages,	M1 phenotype, Bax, cytochrome c↑; M0/M2 phenotype, Bcl-2↓	(34)
Activation of the nitric oxide (NO) pathway	APS	RAW264.7 macrophages, G1 phase arrest	NO, TNF-α, Bax, Bax/Bcl-2 ratio↑; Bcl-2↓	(35)
	GLP	Macrophage, NK cell	NO, Inos, TNF-α、IL-1β, IL-6, IL-10↑	(36, 37)
	*Sargassum fusiforme* polysaccharide SPS	Mitochondria	ROS, P53, Bax, cytochrome c, caspase↑; mitochondrial membrane potential, Bcl-2↓	(38)
Inhibition of cancer cell migration	Kiwi fruit polysaccharide	Macrophages	M1 phenotype↑; PD-1, M2 macrophage markers, tumor volume and weight↓	(39)
	Sea cucumber holothuria tubulosa polysaccharides Ht1 and Ht2	Matrix metalloproteinases	E-cadherin↑; BC MDA-MB-231, MMP-7, MMP-9↓	(40)
	GLP	Reduce phosphorylation of key signaling molecules	ERK1/2, FAK, AKT, Smad2, degradation of TGF-β and EGF↓	(41)
Inhibition of tumor angiogenesis	MAP	Competes with VEGF for binding to VEGFR2	ERK and Akt pathways↑; phosphorylation↓	(42)
	*Dandelion* polysaccharide	Reduce VEGF transcription and secretion	HIF-1α↓	(43)
	Fucoidan, Ht2	Macrophages, matrix metalloproteinases,	Expression of VEGF, MMP-2, MMP-9↓	(40, 44)

Note: ↑ indicates increase/promotion/activation, while ↓ indicates inhibition/reduction/inactivation.

### 2.1 Regulate the immune cells

**Figure 2:**
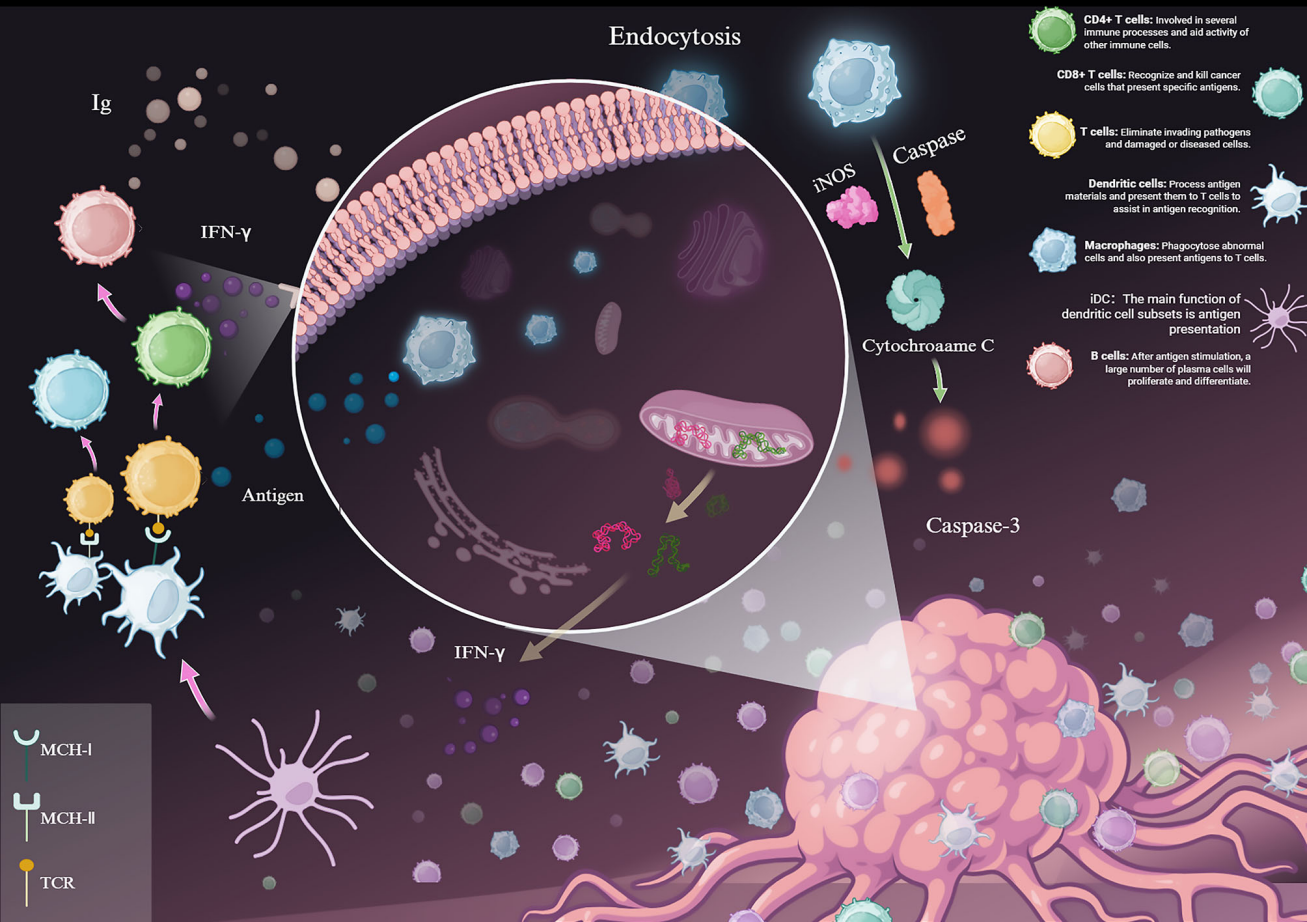
Dynamic changes in the tumor microenvironment. The dynamic interplay of immune cells (including T cells, dendritic cells, macrophages, and B cells) in the tumor microenvironment, highlighting antigen presentation, IFN-γ signaling, and the induction of tumor cell apoptosis via the caspase cascade. (Draw using biorender: https://www.biorender.com/).

Polysaccharides primarily combat tumors by enhancing immune function rather than direct cytotoxicity (45–47). They reverse the immunosuppressive tumor microenvironment by activating various immune cells, including macrophages, dendritic cells, NK cells, and T lymphocytes (48, 49) (**Figure 2**). For example, polysaccharides from Pueraria root stimulate the activate macrophages (RAW 264.7), dendritic cells, NK cells, T and B lymphocytes, and cytokine secretion. This consequently leads to the inhibition of the progression of tumors by acting on multiple mechanisms (8, 50). For instance, yeast-derived β-glucan has been reported to enhance the cytotoxic activity of natural killer (NK) cells against breast cancer cells in both 2D and 3D culture systems (9). Specifically, polysaccharides modulate macrophage polarization (10).

*Polygonatum Sibiricum* polysaccharide (PSP) mediated the TLR4/MyD88 pathway resulting in M2-to-M1 repolarization as shown by the increase in CD86^+^ cells, decrease in CD206^+^ cells, and the consequent anti-hepatoma effect (11). Radix Bupleuri polysaccharide (RBP) contains neutral (RBP-1) and acidic (RBP-2, RBP-3) fractions. Acidic components show stronger macrophage activation. RBP-3 binds TLR2/4, activates MAPK and NF-κB pathways, and alleviates immunosuppression by modulating CD4^+^ T cell differentiation (12). Bacterial exopolysaccharide (EPS) enhances Th1 immunity in colorectal cancer (CRC) models by binding TLR2 on dendritic cells in a MyD88-dependent manner, inducing IL-12 and TNF-α secretion, which promotes T cell-mediated tumor killing (13). *Lentinus edodes* Polysaccharide (LEP) enhances CAR-T cell efficacy in solid tumors by promoting a central memory phenotype (CD44^+^, CD62L^+^, TCF1^+^) and reducing exhaustion markers (TIM-3, CD317) (14). In addition, a study from the United States demonstrated that lentinan modulates gut microbiota composition, increasing the abundance of beneficial bacteria such as Lactobacillus and Bifidobacterium, which in turn enhances systemic anti-tumor immune responses and suppresses colorectal cancer growth in murine models (15). It also repolarizes TAMs to M1 and amplifies ferroptosis via the “IFN-γ-ferroptosis-ROS-Caspase-3 axis”. Combined with iron ions in nano-delivery systems, LEP enhances Fenton-like reactions and remodels the tumor microenvironment (51). LEP also regulates multiple pathways (PI3K/Akt, Wnt/β-catenin, AKT/Nur77/Bcl-2) (52), and immune functions, demonstrating broad anti-cancer potential (53).

Other polysaccharides also exhibit immunomodulatory effects. Fungal heteropolysaccharide TOP60–1 binds TLR2/4, promotes M1 polarization (upregulating CD40^+^, CD80^+^, CD86^+^, MHC-II), and inhibits tumor migration through direct and immunomodulatory mechanism (54). Similarly, polysaccharides isolated from the medicinal fungus Inonotus obliquus (Chaga) have been shown to act as agonists for TLR2 and TLR4 on macrophages, stimulating the secretion of NO, TNF-α, and IL-6, thereby inhibiting cancer cell growth *in vitro* and *in vivo (*16). *Ginseng* polysaccharides (GPS) ameliorates immune organ weight, modulates cytokines (IL-10, TNF-α, IL-6), and enhances PI3K/AKT signaling in S180 sarcoma mice, achieving a 66.52% tumor inhibition rate after 10-day oral administration (17). Lonicera japonica Thunb polysaccharide delivered via exosomes enhances dendritic cell function and strengthens CD8^+^ T cell responses, offering a novel anti-cancer strategy (55). Pectic polysaccharide PEP-1 induces M2-to-M1 transition via NF-κB and MAPK phosphorylation, promoting apoptosis of Hepa1–6 cells *in vitro* and *in vivo (*56).

### 2.2 Inhibit cancer cell proliferation

Polysaccharides inhibit tumor growth by inducing cell cycle arrest at various phases (G0/G1, S, or G2/M), thereby suppressing uncontrolled proliferation (57). For example, a phosphorylated fucoidan-natural product complex upregulates p21, induces G2/M arrest and apoptosis, and suppresses epithelial-mesenchymal transition in oral cancer cells in a dose and time dependent manner (58). Combined with gemcitabine, it synergistically enhances apoptosis and cell cycle arrest in sarcoma models (59). A Hedyotis diffusa polysaccharide induces G0/G1 arrest in Hep2 cells, activates caspases-3, -8, and -9, and downregulates Bcl-2, triggering apoptosis (60). In another study, a polysaccharide from Pleurotus ostreatus upregulates Caspase-9 and Bax, modulates P53, cyclin D, and Cdk4, and induces G0/G1 arrest in Ehrlich ascites carcinoma cells (18). A sulfated galactan isolated from the marine fungus G. fisheri inhibits EGFR/ERK signaling, downregulates cyclin-D, cyclin-E, cdk-4, and cdk-2, and upregulates P53 and p21, leading to G0/G1 arrest in cholangiocarcinoma (19). In addition, a carboxymethylated derivative of laminaran from the brown alga *Saccharina cichorioides* exhibited potent anti-proliferative and anti-invasive activities against human melanoma SK-MEL-28 and colon cancer DLD-1 cells in three-dimensional (3D) cell culture models, highlighting the importance of chemical modification and advanced culture systems for evaluating polysaccharide bioactivity (9). Furthermore, the Safflower polysaccharide significantly reduces Bcl-2 and COX-2, increases Bax and cleaved caspase-3, and induces G0/G1 arrest in tongue squamous cell carcinoma, inhibiting tumor growth *in vitro* and *in vivo (*21).

### 2.3 Promote cancer cell apoptosis

Apoptosis is an autonomous, genetically controlled and ordered cell death process that maintains internal stability (33) (20). Polysaccharides trigger apoptosis-a programmed, genetically controlled cell death-through both intrinsic (mitochondrial) and extrinsic (death receptor) pathways, contributing to their anti-tumor efficacy (22).

HK@PPP-BDBA nanoparticles derived from *Physalis peruviana* polysaccharide induce ROS generation and promote apoptosis in MCF-7 and HeLa cells, while promoting apoptosis cells, resins increasing maturity and anti-tumor immunity (61). Acetylated *Dendrobium huoshanense* polysaccharide activates both mitochondrial and Fas/FasL pathways in HCT116 cells (62). *Ganoderma lucidum* polysaccharides (GLP), is an acid polysaccharide composed of glucose, mannose, galactose, xylose, fructose and arabic (63). GLP selectively prevents pancreatic cell survival, inhibits phage migration through ROS and induces mitochondria death (64). The biological activities of polysaccharides extracted by different methods also change, NaCl-extracted GLP enhances splenocyte proliferation and cytokine secretion, while hot water-extracted GLP promotes B-cell activation (65). Nanoparticles based on Peach gum polysaccharide (PGP) induce mitochondrial apoptosis (Bax, Bcl-2, cytochrome C release) and repolarize macrophages to M1 phenotype, reversing immunosuppression (66). A nanoparticle system constructed from *Lycium barbarum* polysaccharide (LBP) and triptolide significantly reduce mitochondrial membrane potential, increase ROS, and induce efficient apoptosis with low toxicity (67). A homogeneous polysaccharide, IRPS-TE-3 (68) and *Poria cocos* polysaccharide (34) demonstrate anti-apoptotic and immunoprotective activities in non-tumor contexts. Fucoidan activates macrophages via 4-1BB targeting and TNF signaling. This activation indirectly induces apoptosis and G1 arrest in pancreatic cancer cells (69).

Marine algal polysaccharides (MAP) exhibit diverse pro-apoptotic mechanisms (70, 71). A sulfated polysaccharide isolated from the red seaweed *Gracilaria cornea* by a Brazilian research group was shown to induce mitochondrial apoptosis in MCF-7 breast cancer cells through ROS-mediated activation of caspase-9 and caspase-3, while exhibiting low toxicity to normal fibroblasts (72). In breast cancer (BC) cell, MAP induce mitochondrial apoptosis via ROS elevation, lipid peroxidation, and caspase-9/3 activation (23). A water-soluble MAP extracted from *Sargassum* by Digala et al. was shown to induce specific cell death in HeLa cells of cervical cancer (CC), which selectively induces apoptosis in HeLa cells with minimal toxicity to normal cells (24). A homogeneous sulfated polysaccharide, SHA1P-2, promotes TAM apoptosis via CD206-ERK-ROS axis and suppresses T-cell proliferation (25). The sulfated alginate polysaccharide TGC161 inhibits STING-TBK1-IRF3 pathway, reduces T-cell apoptosis, and enhances anti-tumor immunity (26).

A sulfated polysaccharide from the green alga *Caulerpa cupressoides* inhibits melanoma migration and colony formation without inducing apoptosis (27). The anti-tumor mechanism against lung cancer (LC) is more complex. MAP reprogram transcriptome to induce apoptosis and cell cycle arrest in LC cells (28). In contrast, silver nanoparticles synthesized from MAP of fine specula exhibited strong cytotoxicity against A549 cells, while normal cytotoxicity was relatively low (29). On the contrary, porphyria from red algae enhances immune surveillance through indirect anti-tumor effects (30).

### 2.4 Activate the nitric oxide pathway

NO plays a dual role in tumor biology: at high concentrations, it directly induces cancer cell, and it also regulates angiogenesis and immune responses. Polysaccharides often activate macrophages to upregulate inducible NO synthase (iNOS), producing NO that contributes to tumor suppression (31, 32).

For example, although *Astragalus* Polysaccharides (APS) itself has limited direct inhibitory effects on MCF-7 BC cells, conditioned medium from APS-treated RAW264.7 macrophages significantly inhibits cancer cell proliferation (inhibition rate of 41%) and induces G1 phase arrest. Further studies indicate that APS upregulates the expression of NO and TNF-α in macrophages, while also modulating the expression of apoptosis-related genes in cancer cells-upregulating the pro-apoptotic protein Bax and downregulating the anti-apoptotic protein Bcl-2, resulting in a significantly increased Bax/Bcl-2 ratio and initiation of apoptosis (73). Similarly, the GLP (molecular weight 14942 Da) isolated from Colombian radioactive mushrooms not only enhances macrophage proliferation and phagocytosis but also induces the secretion of NO, iNOS, and various cytokines (such as TNF-α, IL-1β, IL-6, and IL-10), thereby exerting synergistic anti-tumor effects (74). The *Grifola frondosa* polysaccharide (GFP) and GLP, have been confirmed as ideal biological response modifiers that enhance both specific and non-specific immune functions. GFP activates the macrophage system and increases NK cell activity, potentially inhibiting tumors through the release of effector molecules like NO (35). GLP mainly regulates immune cells such as macrophages, thereby improving the host immune microenvironment. This regulation indirectly affects the tumor microenvironment. NO may be a key mediator of its anti-tumor mechanism (36).

In addition to immune regulation, NO can also directly induce apoptosis of cancer cells through the mitochondrial pathway. For instance, a novel polysaccharide SPS extracted from *Sargassum fususiforme* can induce apoptosis in human LC A549 cells, accompanied by the loss of mitochondrial membrane potential and the accumulation of reactive oxygen species (ROS). Western blot analysis showed that SPS treatment upregulated the expression of P53 and Bax, down-regulated the expression of Bcl-2, activated caspase-9 and caspase-3, and led to PARP cleavage. This indicates that polysaccharides may activate the caspase cascade by triggering the production of NO or related cellular stress, inducing mitochondrial membrane permeability and the release of cytochrome c, thereby achieving anti-tumor effects (37).

### 2.5 Inhibit cancer cell migration

Cancer cell migration and invasion are key drivers of metastasis and treatment failure. Polysaccharides from diverse sources suppress these processes through multiple mechanisms, targeting adhesion molecules, matrix metalloproteinases (MMPs), and signaling pathways (75).

Polysaccharides from diverse sources share a common ability to suppress cancer cell migration, as demonstrated in multiple experimental systems. Li et al., using *in vivo*, *in vitro*, and in silico analyses, showed that kiwi fruit polysaccharide inhibits migration and invasion of human gastric AGS cells, reduces tumor volume, and remodels the immune microenvironment by downregulating PD-1 and M2 markers while promoting M1 macrophage polarization (38). Capparis ovata polysaccharide suppresses viability and migration of CRC cells (Caco-2, HT-29) by downregulating VEGF and GSK-3β (76). More recently, acidic polysaccharide-enriched extracts from the same sea cucumber species were shown to inhibit the migration and invasion of triple-negative breast cancer cells by upregulating E-cadherin and downregulating MMP-7 and MMP-9, further supporting the anti-metastatic potential of marine-derived polysaccharides (77).

A water-soluble GLP suppresses LC cell viability and migration by reducing phosphorylation of ERK1/2, FAK, AKT, and Smad2, and inducing degradation of TGF-β and EGF receptors, thereby reducing metastatic nodules (39). Among citrus peel polysaccharides, HBE-II most strongly inhibits migration of triple-negative BC cells and angiogenesis by downregulating MMP-9 (78). Laminarin from brown algae inhibits pancreatic cancer cell migration via mitochondrial membrane depolarization, disrupted calcium homeostasis, and suppressed ROS signaling, while showing synergy with 5-fluorouracil (5-FU) (40). Collectively, polysaccharides exert anti-metastatic effects through cell cycle regulation, apoptosis induction, migration/invasion suppression, and immune modulation (41). Sulfated galactomannans, especially low-molecular-weight derivatives, induce G1 arrest, apoptosis, and significantly suppress migration in A549 cells (79).

### 2.6 Inhibit tumor angiogenesis

Therefore, inhibiting tumor angiogenesis has become an important anti-cancer strategy, aiming to “starve” tumors, block metastasis routes, improve immune cell infiltration, and enhance chemotherapy sensitivity. Polysaccharides inhibit tumor angiogenesis through multiple mechanism (80–84). For instance, Liu et al. reported that sulfated polysaccharides from brown algae competitively inhibited VEGF-VEGFR2 binding and blocked ERK and Akt signaling (85). Ren et al. further demonstrated that *Dandelion* polysaccharides down-regulate HIF-1α through PI3K/AKT, reducing the expression of VEGF in the LC model (86). Additionally, citrus peel polysaccharide directly inhibit endothelial tube formation (78). Beyond direct actions on endothelial cells, polysaccharides can indirectly suppress angiogenesis through immunomodulation (87). Fucoidan downregulates VEGF and modulates M2 macrophage polarization (42). Sea cucumber polysaccharide Ht2 suppresses MMP-2/MMP-9, impairing ECM remodeling and angiogenic conditioning (77). Furthermore, a medicinal mushroom heteropolysaccharide simultaneously targets TLR-4 and VEGF, combining immune activation with angiogenesis inhibition (43). LEP elevates IFN-γ and suppresses angiogenesis independently of T-cells (88). A polysaccharide extracted from the roots of Polygala tenuifolia also reduces EGFR, VEGF, and CD34 expression in OC models (44).

**Figure 3:**
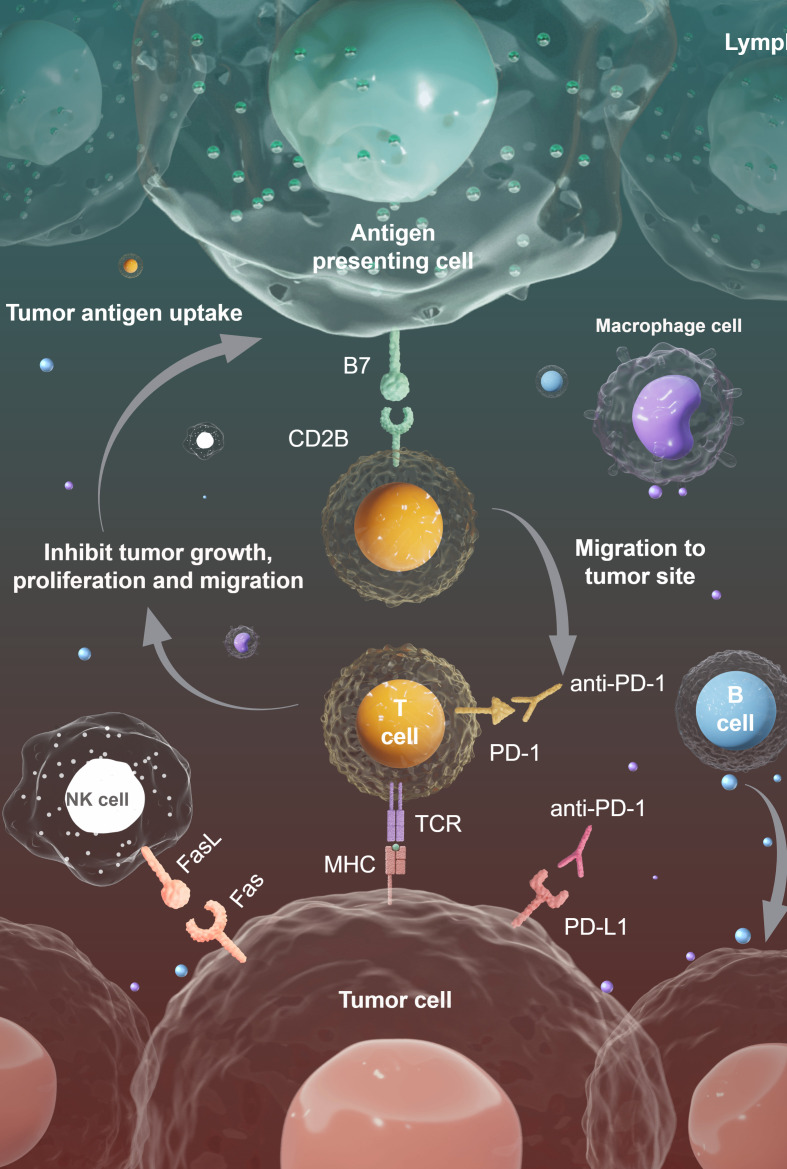
The influence of immune cells on the tumor microenvironment. The antigen-presenting cells activate T cells, while anti-PD-1 therapy blocks PD-1/PD-L1 suppression, enabling T cells, NK cells, and other immune cells to inhibit tumor growth, proliferation, and migration. (Draw using figshare: https://www.figdraw.com/. The authorized ID: UPWTS8969f).

In conclusion, polysaccharides exert anti-tumor effects primarily through immunomodulation, via an intricate network of interconnected cellular and molecular mechanisms. These encompass the activation of immune cells (particularly macrophage M1 repolarization and T cell-mediated responses), induction of tumor cell cycle arrest, triggering of apoptotic cascades via mitochondrial and death receptor pathways, elicitation of NO-dependent cytotoxicity, inhibition of migration and invasion, and suppression of angiogenesis. Their multi-targeted, synergistic modes of action, alongside excellent biocompatibility, render polysaccharides highly promising candidates for both monotherapy and combination anti-cancer therapy (**Figure 3**).

## 3 Polysaccharides modulate tumor metabolism through key signaling pathways

**Figure 4:**
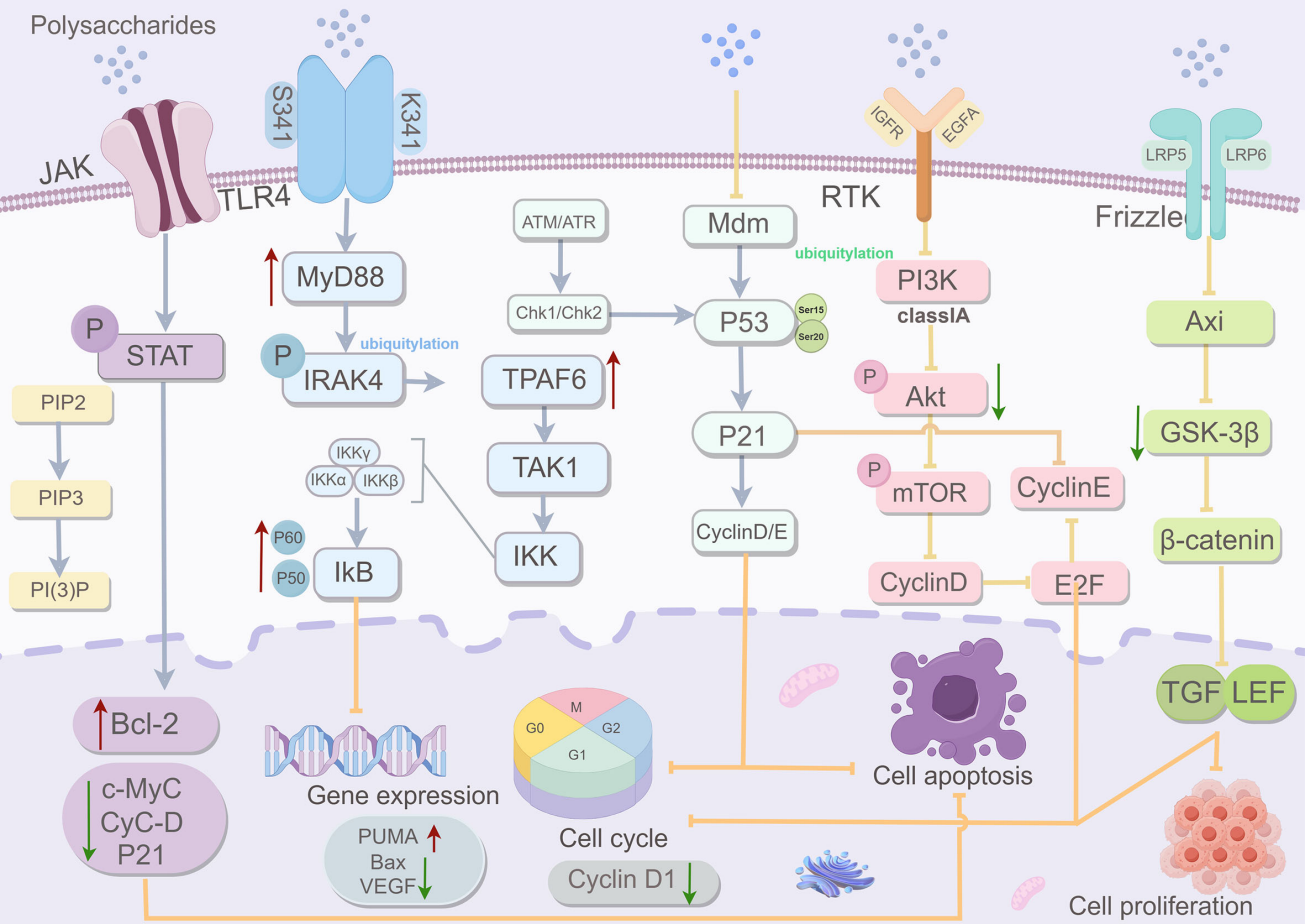
The influence of polysaccharides on tumor-related metabolic pathways. Polysaccharides modulate key oncogenic signaling pathways (TLR4/NF-κB, p53, PI3K/Akt/mTOR, and Wnt/β-catenin) to suppress tumor cell proliferation and promote apoptosis. (Draw using figshare: https://www.figdraw.com/. The authorized ID: RORIIaac1c).

(**Table 2**, **Figure 4**)Polysaccharides exert anti-tumor effects not only through direct immunomodulation and induction of apoptosis (as detailed in Section 2) but also by interfering with the metabolic reprogramming of cancer cells. Tumor cells exhibit altered metabolism, such as enhanced glycolysis (Warburg effect), increased glutamine consumption, and elevated lipid synthesis-to support uncontrolled proliferation. Emerging evidence indicates that polysaccharides modulate multiple signaling pathways, including P53, NF-κB, Wnt/β-catenin, PI3K/Akt, TLR, and Fas/FasL-ROS/JNK-that are intimately linked to these metabolic processes. This section highlights how polysaccharides, through these pathways, reshape tumor metabolism to suppress proliferation, induce cell death, and enhance anti-tumor immunity.

**Table 2:** The anti-cancer effects of polysaccharides from different sources.

Source	Name	Monosaccharide composition	Cancer	Mechanism.	Ref.
*Astragalus membranaceus*	*Astragalus* Polysaccharides (APS)	Glucose, rhamnose, xylose, mannose, glucuronic acid, arabinose, and galactose	Lung cancer cells (LC, A549, NCI-H358), bladder cancer cells, ovarian cancer (OC) cells, cervical cancer cells (HeLa), breast cancer (BC) cells (MCF-7), gastric cancer (GC) cells (MGC-803), sarcoma S180 cells	P65, P50, gene FBXW7, TNF-α, IFN-γ, FoxO1, E-cadherin and IL-2 ↑. FoxO1, MMPs1, PI3K-p110β, phosphorylated AKT levels, CCND, miR-27a, PPARD/CDC20 axis, Wnt, β-catenin, TCF, N-cadherin/Vimentin↓	(89, 90)
*Ginseng*	*Panax* polysaccharides/*Ginseng* polysaccharides (GPS), *Pseudostellaria heterophylla* polysaccharides	Glucose, rhamnose, xylose, mannose, glucuronic acid, arabinose, and galactose	sarcoma S180 cells, colorectal cancer (CRC), Hepatic carcinoma (HC) cells, GC cells (HGC-27), LC and B16F10 melanoma	TNF-α, TNF, IL-6, PI3K, AKT, Ca²^+^, M2-type macrophages ↑. IL-10, phosphorylation level of serine/threonine protein kinase B, G0/G1 cell phase, Twist and AKR1C2, G2/Mcell phase, M1-type macrophages↓	(91)
*Dendrobium nobile Lindl*	*Dendrobium* polysaccharides	Mannose, glucose, galactose, xylose and acetyl modifications	HCT116, BC, HC cells GC, non-small cell lung cancer (NSCLC)	Caspase-3/8, NLRP3, Bax/Bcl-2 ratio, ROS, JNK, Fas-FasL ↑ Bcl-2, UDP-GlcNAc, ST6Gal-I, IL-35, G2/M cell phase↓	(92–94)
*Lycium barbarum L.*	*Lycium barbarum* polysaccharides (LBP)	Glucose, mannose, galactose, arabinose, xylose, fucose and rhamnose	MCF-7, human cutaneous squamous cell carcinoma A431 cells, MGC-803, SGC-7901, human hepatoma cell line QGY7703, SW480 cells, Caco-2 cells and A549 cells	Ki67 and PCNA, cl-caspase-3, caspase-3/7, miR-202-5p ↑ SLC7A11, GPX4, Bcl-2, LC3II, ERK1/2, PIK3CA, AKT and mTOR ↓	(95–105)
*Fungi*	*Ganoderma lucidum* polysaccharides (GLP), Inonotus obliquus polysaccharides (AcF1, AcF3), lentinan, cordyceps	Glucose, mannose, galactose, xylose, fructose and arabinose	PANC-1 cells, BxPC-3 cells, Mia PaCa-2 cells, MG-63, UC, MCF-7 HeLa, 786-O, SKOV3 cells and LC cells, HT29 cell, SW480 cell, heLa cells	ROS, NO, IL-6, TNF-α, IL-1β, B-cell activity↑; Bcl-2, TLR2, TLR4, cyclin E, cyclin A, CDK2, S-phase↓	(106–110)
Algae	Marine algal polysaccharides (MAP); Sulfated polysaccharides, *Sargassum fusiforme* polysaccharide (SFP2205), fucoidan	AGAR, carrageenan and alginate, etc	HeLa, A549 cells, MCF-7, MDA-MB-231, B16-F10 (melanoma), A549, 4T1 BC cells, OC (SKOV-3, A2780), pancreatic cancer	ROS, MDA, Caspase-9 and Caspase-3↑ mitochondrial, phosphorylated IRF3, STING-TBK1-IRF3, G1 cell cycle, β-catenin, T-cell, c-Myc, cyclin D1, survivin↓	(26, 30, 31, 111)
*Portulaca oleracea*	*Portulaca oleracea L*. polysaccharides	Uronic acid, galactose, glucose, arabinose, xylose, and minor rhamnose.	Sacroma 180 cells, heLa cells, CC cells, CTT-116 cells	TNF-α, IL-6, IL-10, TLR4, MyD88, NF-κB↓ IFN-α, WBC, MPO, MDA, miR-146a, miR-1et 7↑	(39–41, 78, 79)
*Balanophora polyandra*	*Balanophora polyandra* polysaccharides		OC	P53 mRNA, cancer cell invasion and migration↑, S-phase↓	(101)
Chrysanthemum	Echinacea polysaccharides, flowers of Inula japonica (IJP70-1)		LC	Propionate, butyrate↑; TLR4, NF-κB, IL-6, MMP-2↓	(112, 113)
*Cyclocarya paliurus*	*Cyclocarya paliurus* polysaccharides (CM-CP, S-CP)			TNF-α, NO, Ca²^+^↑; NF-κB, Na^+^/K^+^-ATpase and Ca²^+^-ATpase activities↓	
*Homogeneous*	*Homogeneous* polysaccharides		HC	Mitochondrial dependent apoptosis↑; Wnt, β-catenin, G2/M phase↓	(114)
*Atractylodes macrocephala*	*Atractylodes macrocephala* polysaccharides		CRC, LC cell	IL-6, IFN-γ, TNF-α, NO↑; TLR4↓	(115)
*Polygonatum Sibiricum*	PSP	111, glucuronic acid, rhamnose, galacturonic acid, glucose and arabinose	HC, LC	NO, IL-6, IFN-γ, MAPK↑; TLR4 mRNA↓	(116, 117)
Peach Gum	Peach Gum polysaccharide (PGP)		CRC (CT-26 cells), ulcerative colitis	IL-6、TNF-α↑; PI3K, Akt↓	(34, 118)

Note: ↑ indicates increase/promotion/activation, while ↓ indicates inhibition/reduction/inactivation.

### 3.1 Activate the P53 signaling pathway

P53, a critical tumor suppressor, maintains genomic integrity and prevents tumorigenesis by inducing cell cycle arrest, apoptosis, and inhibiting angiogenesis. The tumor suppressor P53 is a master regulator of both cell fate and metabolism. Beyond inducing cell cycle arrest and apoptosis, P53 controls metabolic pathways by suppressing glycolysis and promoting oxidative phosphorylation. For instance, P53 downregulates glucose transporters and inhibits glycolytic enzymes, while it enhances mitochondrial respiration through the synthesis of cytochrome c oxidase 2. It also regulates glutamine metabolism via glutaminase 2, shifting cells toward oxidative utilization of glutamine.

Polysaccharides from various plants and fungi exert anti-tumor effects by regulating P53 expression. For instance, GLP activates the P53 protein in CRC HT29 and SW480 cells, inhibiting proliferation and promoting apoptosis through transcription-dependent and independent mechanisms (119). This finding aligns with studies showing that GLP show prominent anticancer activities by reactivating several types of mutant p53 (120).

Furthermore, in both *in vitro* and *in vivo* OC models, polysaccharides extracted from *Balanophora polyandra* increase P53 mRNA and protein levels in ovarian cancer models, leading to S-phase arrest (121). Similarly, *Cordyceps sinensis* polysaccharides upregulate P53 expression in HeLa cells, inhibit cyclin E, A and CDK2, induce S-phase arrest, and ultimately lead to cell cycle arrest and apoptosis. These studies suggest that P53-mediated metabolic reprogramming contributes to the overall anti-tumor efficacy. Future investigations should directly assess how polysaccharide-induced P53 alters the metabolic landscape of cancer cells (122).

### 3.2 Inhibit the NF-κB signaling pathway

NF-κB is a nuclear transcription factor that regulates cellular responses to stimuli, including inflammation and immunity. In cancer, NF-κB promotes proliferation, apoptosis resistance, angiogenesis and immune surveillance (106, 107, 123). *Pseudostellaria heterophylla* polysaccharides (PHP-1) reprograms TAMs via TLR4-mediated Ca²^+^ release and NF-κB/MAPK pathway activation, promoting M2-to-M1 repolarization (106). Research from India has reported that *Aloe vera* polysaccharides suppress the proliferation of A549 lung cancer cells by inhibiting the NF-κB signaling pathway and reducing the expression of pro-inflammatory cytokines IL-6 and TNF-α (124). Echinacea polysaccharides are metabolized by gut microbiota to produce SCFAs that inhibit TLR4/NF-κB signaling, reducing IL-6 and MMP-2 expression in LC (125). APS at exhibits broad anti-cancer activity in A549 and NCI-H358 cells (126) by inhibiting NF-κB transcriptional activity in LC despite upregulating P65/P50 (127), inducing G0/G1 arrest in bladder cancer via CCND1 downregulation (128), suppressing ovarian cancer (OC) through miR-27a/FBXW7 axis (129), inhibiting CC via PPARD/CDC20 regulation (129, 130), arresting BC cells in G1 phase (73). Modified polysaccharides, isolated by Yu et al., show enhanced efficacy, ASP4 fraction induces apoptosis in GC and sarcoma while modulating cytokines (131), elevated serum levels of TNF-α, IFN-γ, and IL-2, while reducing IL-6 (89). Carboxymethylated/sulfated *Cyclocarya paliurus* polysaccharides activate RAW264.7 cells via NF-κB, inducing mitochondrial dysfunction and Ca²^+^ overload (132). These findings highlight polysaccharides as multi-target agents modulating NF-κB for cancer therapy.

### 3.3 Inhibit Wnt signaling pathway

Aberrant activation of the Wnt/β-catenin pathway drives malignant proliferation by transcriptionally activating c-Myc and cyclin D1. c-Myc, in turn, orchestrates a broad metabolic program that includes increased glutamine uptake, glutaminolysis, nucleotide synthesis, and lipid biogenesis. Thus, targeting Wnt/β-catenin can impose metabolic stress on cancer cells. Similarly, a polysaccharide derived from okra flowers also exhibits anti-CRC activity via regulation of this pathway (133). Sulfated brown algal polysaccharides suppress β-catenin/TCF activity, downregulating c-Myc/cyclin D1/survivin in BC (134). *Dendrobium* polysaccharides concurrently inhibit Wnt/β-catenin and PI3K/AKT/mTOR pathways via ROS/JNK activation, thereby targeting hepatocellular carcinoma (HCC) cells. Additionally, low-molecular-weight fragments (<5 kDa) amplify ROS signaling to induce G2/M arrest (135). *Dendrobium officinale* Polysaccharides (DPS) can inhibit the occurrence and development of GC by regulating the Wnt/β-catenin pathway and altering endogenous metabolites (136). Folic acid-conjugated DPS nanoparticles were used for targeted delivery to solid tumors, resulting in tumor cell death (137).

**Figure 5:**
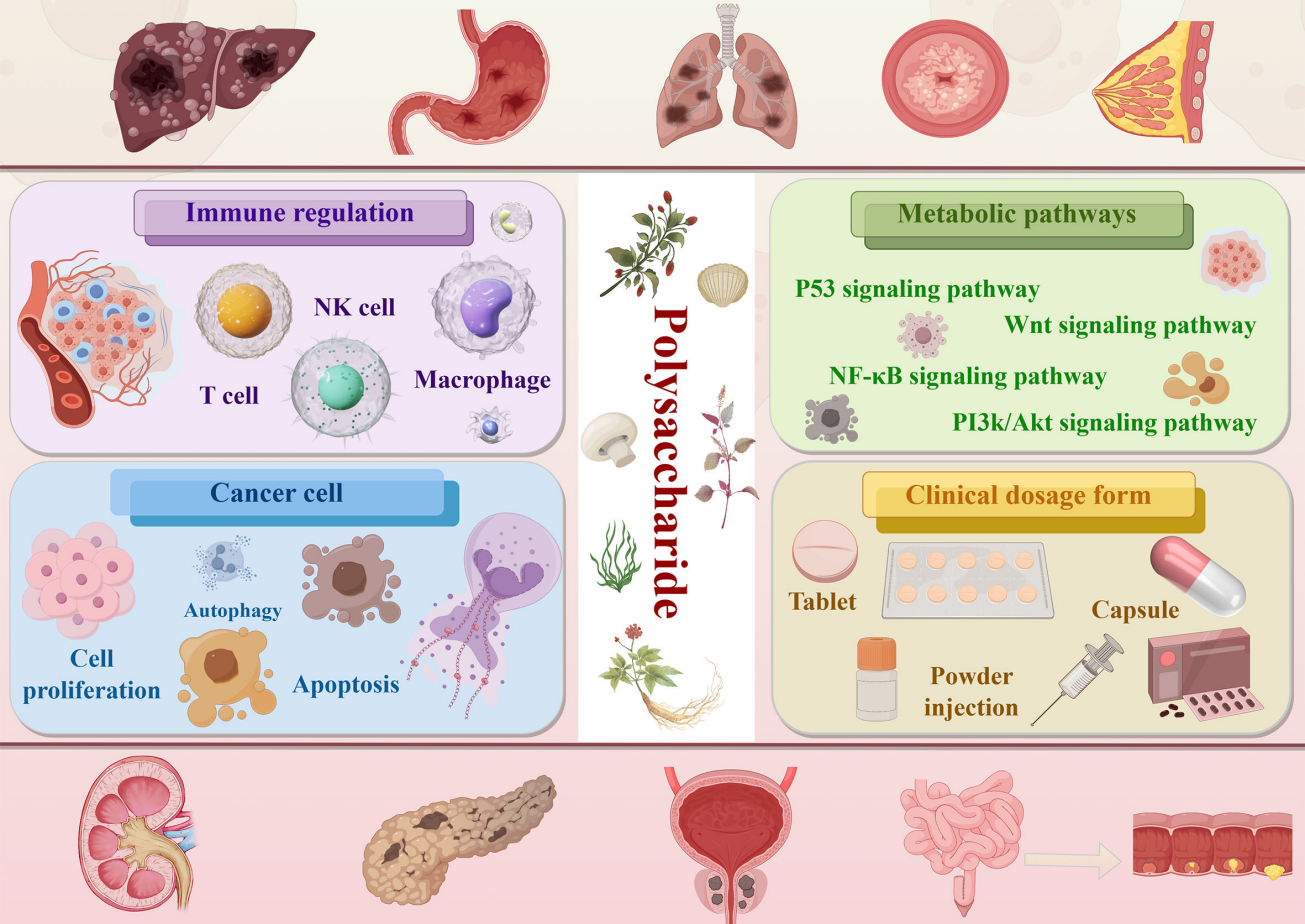
Graphical abstract: The mechanism of action and clinical application forms of polysaccharides in cancer treatment. The polysaccharides anti-cancer mechanisms (immune regulation, cancer cell inhibition, signaling pathway modulation) and clinical dosage forms of polysaccharides.(Draw using figshare: https://www.figdraw.com/. The authorized ID: OUASPce089).

APS suppresses Wnt signaling through: β-catenin/TCF inhibition and EMT reversal in BC, PPARD/CDC20 axis regulation in CC (138). In CRC, *Gynostemma pentaphyllum* leaf polysaccharide blocks β-catenin nuclear translocation in colon cancer (139). Recent research on ASP regulates miR-3187-3p/PCDH10 axis to anti- BC (140). Multiple reviews on LBP regulate Wnt/β-catenin via PI3K-Akt-GSK-3β crosstalk (141).

Polysaccharides can also indirectly inhibit Wnt pathway through the Galectin-3 axis. Galectin-3 can promote β-catenin nuclear accumulation and enhance TCF transcriptional activity (142). Modified citrus pectin, a natural Galectin-3 antagonist, downregulates β-catenin signaling, establishing a polysaccharide-Galectin-3-β-catenin inhibitory axis. This pathway intersects with inflammation/STAT3 and PI3K/Akt networks, suggesting polysaccharides disrupt oncogenic homeostasis (143).

### 3.4 Inhibit the TLR signaling pathway

Toll-like receptors (TLRs) are innate immune sensors that not only trigger inflammatory responses but also profoundly influence cellular metabolism. Activation of TLRs on macrophages induces a metabolic switch toward aerobic glycolysis (similar to the Warburg effect), which supports the rapid production of cytokines and antimicrobial effectors. In the tumor microenvironment, appropriate TLR stimulation can reprogram immune cells to a pro-inflammatory, anti-tumor state, and this metabolic shift is integral to their function. Polysaccharides often act as TLR agonists to exert anti-tumor effects. Research indicates that *Atractylodes macrocephala* polysaccharide is recognized by TLR4 on macrophages, activating the TLR4/MyD88 signaling pathway. This upregulates the expressions of IL-6, IFN-γ, TNF-α and NO, enhances their phagocytic ability against CRC cells, thereby inhibiting the progression of CRC. The absence of this anti-tumor effect in TLR4 knockout mice further confirmed the key role of TLR4 in this pathway (92). A similar TLR4-dependent anti-tumor mechanism was also verified in a mouse LC model treated with cocoa polysaccharides. In PSP treated LC cells (11, 93, 94). PSP exerts its anti-tumor effect by regulating the TLR4-MAPK/NF-κB pathway. It up-regulates mRNA and protein expression at key signaling nodes, promotes NO and cytokine secretion, improves immune organ indices (90).

In addition, the homogeneous polysaccharide IJP70–1 purified from Japanese chrysanthemum interacts with TLR4, PD-1 and VEGF proteins, activates the immune system, inhibits tumor angiogenesis, and ultimately inhibits tumor proliferation (144). Two water-soluble polysaccharides, AcF1 and AcF3, isolated from the medicinal fungus Inonotus obliquus can act as agonists for the TLR2 and TLR4 receptors of macrophages. They stimulate the secretion of NO, TNF-α and IL-6, and work in synergy with IFN-γ to inhibit the growth of cancer cells, showing great potential for cancer immunotherapy both *in vitro* and *in vivo (*16). Furthermore, studies have shown that lentinan, as a drug carrier, affects the endocytosis process through the interaction between TLR4 and CAV1. This process is regulated through the TLR4/TRAF3/MFN1 signaling pathway, guiding drugs into tumor mitochondria and providing a new strategy for the treatment of malignant ascites (145).

### 3.5 Inhibit PI3k/Akt signaling pathway

The PI3K/Akt pathway is a central hub for integrating growth factor signals with cellular metabolism. Akt promotes glucose uptake by translocating GLUT1 to the plasma membrane, activates hexokinase to trap glucose as glucose-6-phosphate, and stimulates lipid synthesis via SREBP transcription factors. It also suppresses oxidative phosphorylation and enhances glycolysis, thereby supporting the anabolic needs of proliferating cancer cells (146, 147). Polysaccharides inhibit this cascade to exert anti-tumor effects. APS induces apoptosis of bladder cancer cells by down-regulating CCND1, inhibiting PI3K-p110β/p-Akt, up-regulating FoxO1, and inducing G0/G1 arrest (128). *Dandelion* polysaccharide blocks PI3K/Akt/mTOR signaling, causing G0/G1 arrest in hepatocellular carcinoma without affecting normal cells (148). GPS, from *Panax Ginseng C. A. Mey*, can be classified into neutral or acidic pectins (149). Component GP-A activates IL-6-mediated PI3K/AKT in splenic immune cells (17), while other fractions inhibit PI3K/AKT in CRC (150). Purified PGP2a inhibits human GC HGC-27 cells dose-dependently, suppressing Twist and AKR1C2, and enhancing apoptosis and G2/M arrest (151, 152). GPS enhances PD-1/PD-L1 therapy efficacy in LC models (153). SFP2205, modulates Caspase-9 and Caspase-3 to block PI3K/AKT, inhibiting human erythroleukemia growth (154). Furthermore, a recent review by Cancemi et al. (2024) comprehensively summarized the role of *Ganoderma lucidum* polysaccharides in modulating the PI3K/AKT/mTOR pathway and inducing apoptosis in various cancer types, reinforcing the therapeutic potential of mushroom-derived polysaccharides in oncology (155). PGP promotes epithelial proliferation via PI3K/AKT while protecting intestinal barrier function and modulating the tumor immune microenvironment in CRC (156).

These findings establish polysaccharides as multi-functional inhibitors of the PI3K/Akt pathway, exerting their therapeutic effects by inducing apoptosis, arresting the cell cycle, suppressing metastasis, and modulating the tumor microenvironment.

### 3.6 Inhibit Fas/FasL pathway and the ROS/JNK signaling pathway

The Fas/FasL (extrinsic) and ROS/JNK (stress-induced) pathways are traditionally associated with apoptosis, but they also intersect with metabolic regulation. Reactive oxygen species (ROS) can damage mitochondrial DNA and electron transport chain complexes, impaired oxidative phosphorylation and forcing cells to rely on glycolysis. Excessive ROS also trigger the unfolded protein response and autophagy, leading to metabolic catastrophe. JNK signaling can modulate insulin sensitivity, lipid metabolism, and autophagy, further influencing cellular energetics.

Polysaccharides activating these pathways may induce metabolic dysfunction. *Dendrobium* polysaccharides (DPS) activate the Fas/FasL pathway, downregulating Bcl-2 and upregulating cleaved caspase-3/8 in colorectal cancer, concurrently, they may enhance ROS production, disrupting mitochondrial membrane potential and ATP synthesis (135, 157). Acetylated DPS disrupts glycosylation in HCT116 cells by inhibiting UDP-GlcNAc and ST6Gal-I (62). In the tumor microenvironment, DPS blocks IL-35-mediated differentiation of iTr35 cells, synergizing with ROS/JNK activation to suppress non-small cell lung cancer (NSCLC) progression (158, 159).

LBP induces ferroptosis in BC via xCT/GPX4 pathway inhibition and mitochondrial dysfunction (160). LBP inhibits cutaneous squamous cell carcinoma by down-regulating the expressions of Bcl-2, LC3II and p-ERK1/2 (161). LBP can suppress gastric cancer (GC) proliferation via caspase-3/7 activation and miR-202-5p-mediated PI3K/AKT/mTOR inhibition (162). Further studies have shown that LBP has a significant inhibitory effect on human GC MGC-803 and SGC-7901, inducing G0/G1 arrest (163). Zhang et al. found that LBP inhibits proliferation of human hepatoma QGY7703 cells by inducing S-phase arrest (164). It also induces G0/G1 arrest in CRC cells (91) and selectively causes G2-phase arrest in A549 cells (165).

LEP enhances pemetrexed sensitivity in NSCLC by inhibiting PI3K/Akt and elevating ROS (166); activates the EGR1/PTEN axis in hepatocellular carcinoma, promoting Bax/Bcl-2-mediated apoptosis (167).

In summary, polysaccharides modulate multiple critical signaling pathways-p53-mediated cell cycle checkpoint, NF-κB-driven inflammation, Wnt/β-catenin-dependent proliferation, TLR-mediated innate immunity, PI3K/Akt/mTOR pro-survival signaling, and Fas-FasL/ROS/JNK-triggered apoptosis. Their coordinated, multi-target effects underpin broad-spectrum anti-tumor activity with favorable safety profiles.

## 4 Selective cytotoxicity of polysaccharides

One notable characteristic of polysaccharides is their selective cytotoxicity: they can effectively kill cancer cells while not harming normal cells. This selectivity stems from the differences in cell membrane properties, receptor expression, and metabolic status between malignant cells and non-malignant cells, and is manifested in extremely high selectivity indices and minimal off-target effects. Numerous examples have demonstrated this phenomenon.

PEP-2 from *Pleurotus citrinipileatus* induces apoptosis in HepG-2 cells via chromatin condensation without affecting normal cells (168). Boletus mushrooms polysaccharide inhibits LS180 CRC cells *in vitro (*169) and suppresses renal carcinoma *in vivo* without hematological or organ toxicity (170). Rosa laevigata fruits heteropolysaccharide JYP70-1 (Mw: 1.90×10^4^ g/mol) inhibits tumor growth and metastasis concentration-dependently with low systemic toxicity (118). *Althaea officinalis* polysaccharide AMPS-a contains a β-d-Glcp (1→3) α-Fucp branch that may facilitate membrane penetration and contribute to cytotoxicity, though the precise mechanism remains unclear (171). Low-MW chitin derived from the shrimp strongly inhibits human monocytic leukemia THP-1 cells(IC_50_ of 1 μg/ml), an effect comparable to that of 5-FU (IC_50_ = 0.606 μg/ml), This is attributed to electrostatic interactions with negatively charged tumor membranes (172, 173).

*Antrodia cinnamomea* sulfated polysaccharide N50F2 triggers ER stress-mediated apoptosis in LC via CHOP/caspase-3 and AKT/ERK/EGFR activation, and suppressing the expression of downstream targets such as Slug, TGFRI, and TGFRII, sparing normal cells (95, 96). GLP-5-FU conjugates demonstrate enhanced drug release and cytotoxicity, significantly inhibiting HeLa, 786-O, and SKOV3 cells while showing low toxicity to normal 293A cells (97).

GLP can also inhibit osteosarcoma (MG-63) (98), urothelial carcinoma (99), and BC cell line (MCF-7) (100), highlighting its multi-target potential. Lactobacillus exopolysaccharides (EPS) exhibit selective anti-tumor activities through apoptosis induction, cell cycle arrest, anti-angiogenesis, and anti-inflammation (101), EPSF1 inhibits Caco-2 cells (IC_50_: 72.5 μg/mL) while preserving WI-38 normal cells (102, 103). *Pediococcus pentosaceus* ESP-2 inhibits HCT116 proliferation without damaging intestinal epithelial cells (104).

Fucoidan exemplifies pronounced selectivity. It suppresses proliferation, induces G0/G1 arrest, and promotes apoptosis in human OC cells (SKOV-3, A2780) with IC_50_ values of 82-155 μg/mL, whereas the IC_50_ for normal ovarian epithelial IOSE80 cells is 393 μg/mL (selectivity index >4), with no significant apoptosis or cell cycle arrest in normal cells (105). Fucoidan (100-400 μg/mL) significantly inhibits proliferation and metastasis of 4T1 BC cells without markedly affecting mouse splenocytes or bone marrow cells (174). Immobilized fucoidan on fibrous meshes shows toxic effects on melanoma cells but not on non-cancer skin cells (175). Low-molecular-weight fucoidan polarizes macrophages toward M1 via TLR4/NF-κB and MAPK p38 pathways, inhibiting pancreatic cancer PANC-1 cells (>60% inhibition) while minimally affecting HUVECs (viability >85%) (176). The selective cytotoxicity of a *Ganoderma lucidum* ethanol extract against BC cells was significant. It exhibited an IC_50_ of 62.37 μg/mL on MCF-7 cells, whereas the IC_50_ on normal breast MCF-10A cells exceeded 1000 μg/mL (cell survival rate >90%), yielding a high selectivity index (>16). This extract exerts anticancer effects by inducing G0/G1 phase arrest, downregulating energy metabolism genes (ACAT1, ADCY3, NME2), and activating the mitochondrial apoptosis pathway (downregulating Bcl-2, upregulating Bax and Caspase-9), while showing no significant toxicity to normal cells (112).

Nanotechnology-based formulations further enhance this selectivity. For instance, melatonin-loaded leucolipid-chitosan nanoparticles (NP-MEL) displayed a CC_50_ of 109.53 μg/mL against 4T1 breast cancer cells, compared to 1460.59 μg/mL against normal VERO cells (selectivity index: 13.33). *In vivo* studies on BALB/c tumor-bearing mice treated with NP-MEL (2 mg/kg/day for 21 days) revealed no significant changes in body weight, clinical signs, or liver/kidney function biomarkers, with histopathological analysis confirming normal organ architecture. In contrast, the blank nanoparticle control group showed moderate to severe renal injury (177).

Another example is lactoferrin-oleic acid complex-loaded chitosan nanoparticles (cLf-OA and hLf-OA) demonstrated significantly lower IC_50_ values against cancer cells (HepG-2, Caco-2, HeLa, MCF-7) than against normal WI-38 cells, a selectivity not observed with the uncomplexed forms. This indicates that nanoencapsulation reduces normal cell toxicity while enhancing cytotoxicity and apoptosis induction in cancer cells (178). Chitosan-coated silver nanoparticles (AgNPs-CHI) exhibited potent cytotoxicity against MCF-7 cells, with toxicity to normal human skin fibroblasts (HSF) being 10-fold lower. This formulation reduced IL-6 and TNF-α levels in MCF-7 cells by 90% and 30%, respectively, compared to 60% and 10% with ordinary AgNO_3_, demonstrating superior selectivity and anti-inflammatory activity (155).

Collectively, a growing body of evidence demonstrates that polysaccharides exhibit marked selective cytotoxicity-efficiently killing diverse cancer cells (e.g., liver, colorectal, leukemia, ovarian, breast, lung) while sparing normal counterparts. This selectivity is reflected in high ratios, minimal off-target effects, and differential expression of pattern recognition receptors (e.g., TLR4) and metabolic vulnerabilities between tumor and normal cells, and is attributed to distinct membrane properties, receptor expression profiles, and metabolic states in malignant versus non-malignant cells. The application of nanotechnology (such as chitosan nanoparticles, liposomes, and polymer micelles) further enhances this selectivity by optimizing drug delivery, controlling release, and targeting modification (such as folate receptor targeting), while reducing system toxicity and improving the therapeutic window. These characteristics position polysaccharides as promising candidates for cancer therapy with reduced side effects compared to conventional chemotherapeutics.

## 5 The relationship between polysaccharide structure and anti-tumor properties

(**Table 3**)The biological activity of polysaccharides is highly dependent on their structural characteristics, that is, the structure-activity relationship. Anticancer activity, as one of the important biological activities of polysaccharides, is related to multiple dimensions such as the composition of monosaccharides, MW, connection mode of glycosidic bonds, and spatial conformation. Currently, a large number of studies have systematically revealed the intrinsic regulatory laws between the various structural characteristics of polysaccharides and their anticancer activity.

**Table 3:** The structural characteristics of polysaccharides and the structure-activity relationship of their anti-cancer properties.

Structure - Anticancer Relationship	Monosaccharide composition (core basic structure)	Molecular weight (core physicochemical parameters)	Glycosidic Linkages (Advanced structural support)	Spatial conformation (advanced structural core)	Chemical modification (aimed at enhancing anti-cancer activity)
Core regulatory function	The structural framework foundation that determines the anti-cancer activity, it is the fundamental prerequisite for the formation of active sites and the distribution of charges, and regulates the basic binding ability of polysaccharides with tumors/immune cells.	Governs the transport, distribution, and metabolism of polysaccharides *in vivo*, and regulate the mode of realization of the anti-cancer effect (direct/indirect) and bioavailability	Determine the main branch chain structure of polysaccharides, which is the structural basis for the formation of spatial conformation, and regulate the stability of the active conformation and the exposure degree of the active site	Polysaccharides’ specific binding to target molecules is a crucial prerequisite, directly determining the initiation efficiency of the anti-cancer effect and the receptor recognition ability.	① Alter the charge distribution of polysaccharides (sulfation and phosphorylation introduce negative charges, methylation regulates charge density); ② Regulate the balance of molecular hydrophobicity (acetylation enhances hydrophobicity); ③ Stabilize the active spatial conformation (methylation and sulfation strengthen the hydrogen bond network); ④ Optimize the exposure efficiency of active sites
Optimal structural features	Use β-D-glucose as the backbone, containing an appropriate ratio of acidic monosaccharides (galacturonic acid/glucuronic acid), arabinose; D-type chiral configuration, moderately sulfated/acylated modification, moderate branching degree; The molar ratio of active monosaccharides is within the threshold range	There exists an optimal suitable range (10–100 kDa), with uniform molecular weight distribution; The low molecular weight segment (< 10 kDa) is suitable for direct intracellular action, while the medium-high molecular segment (10–100 kDa) is suitable for immune regulation	The core is a β-type glycosidic bond (especially β-1,3-glycosidic bond), supplemented by a small amount of β-1,6-glycosidic bond branches; The branching degree is controlled at 0.2 - 0.5, and the bonding position is preferably 1,3/1,6 combination.	The triple helix conformation is the optimal; The conformation has good rigidity and can stably exist under physiological conditions (normal pH/ion strength), and the active site is fully exposed.	① Degree of substitution (DS): Sulfation 1.0 - 2.0, Acetylation 0.5 - 1.2, Methylation 0.8 - 1.5, Phosphorylation 0.6 - 1.8. All of these need to be controlled within an appropriate range; ② Group distribution: Sulfate and phosphate groups are evenly distributed on the main chain, while acetyl groups are modified at adjacent positions of the branches or active sites; ③ Appropriate polysaccharide type; ④ Modification site: Avoid the key positions of the main chain glycosidic bonds.
Main anti-cancer mechanism	① Activate pattern recognition receptors of immune cells, promoting the release of anti-tumor cytokines (TNF-α/IFN-γ); ② Achieve targeted binding through specific receptors on tumor cell surface, blocking nutrient intake and inhibiting migration and invasion; ③ Enhance water solubility and transmembrane transport efficiency, regulating the acid-base balance of the tumor microenvironment; ④Sulfation/acetylation modification enhances immune activation and targeting binding ability.	① Low molecular weight: Penetrate the cell membrane to directly regulate the apoptosis pathway of tumor cells (inhibit PI3K/Akt, upregulate Caspase-3), eliminate ROS in the tumor microenvironment; ② Medium-high molecular weight: Activate antigen-presenting cells, reshape the immunosuppressive microenvironment, activate the complement lytic pathway; ③ Uniform molecular weight enhances the stability of target binding.	① The structure mainly composed of β-1,3-glycosidic bonds forms a triple helix conformation, binding to the Dectin-1 receptor to activate the NF-κB/MAPK signaling pathway; ② Moderate branching increases the active site, improving the target binding efficiency; ③ α-type glycosidic bonds have unstable conformation, resulting in weak binding ability.	① The triple helix conformation is easily recognized by immune cell receptors, has strong anti-enzymatic degradation ability, and prolongs the duration of action in the body; ② Maintain conformational stability through intramolecular hydrogen bonds/static forces, ensuring continuous binding with the target molecule; ③ Chemical modification (methylation/sulfation) can enhance conformational stability, further enhancing activity.	① Activation of immune regulatory pathways; ② Direct tumor suppression effect; ③ Regulation of tumor microenvironment; ④ Inhibition of migration and invasion
Key Characteristics of Activity	The structural foundation determines the “lower limit” of activity, while the modification method and proportion regulate the “upper limit: of activity; different sugar combinations achieve the synergy of targeting and immunological activity.	Activity has a non-linear relationship with molecular weight, without simple positive/negative correlation; the molecular weight can be regulated through artificial degradation/modification to optimize activity.	It is highly coordinated with the spatial conformation, providing structural support for the conformation, and its characteristics directly determine the activity value of the conformation	It is the external manifestation of the structural characteristics of glycosidic bonds, and the disruption of the conformation will directly lead to a significant loss of anti-cancer activity; it is significantly affected by external environments (pH/temperature)	① Degree of substitution dependence: shows non-linear correlation; ② Structural compatibility: acidic polysaccharides are mostly compatible with sulfation, while neutral polysaccharides are mostly compatible with phosphorylation; ③ Differences in action modes: sulfation/phosphorylation mainly exert immunomodulatory effects, while acetylation mainly has direct tumor-suppressing effects; ④ Synergistic and additive effects; ⑤ Tumor specificity: has inhibitory effects on various solid tumors.
Ref	(108–110, 114, 179–181)	(182–185)	(113, 115–117, 186–191)	(111, 192–195)	(196–207)

### 5.1 Monosaccharide composition

The monosaccharide composition of polysaccharides is the primary structural feature that determines their anti-cancer activity. Neutral monosaccharides (such as glucose, galactose, and mannose) are significantly associated with anti-cancer activity (208), while acidic sugars (galacturonic acid, glucuronic acid) further enhance activity by increasing water solubility and charge density (179). For example, in pectin polysaccharides, a high content of galacturonic acid can form homogalacturonan and rhamnogalacturonan-I domains, enhancing specific binding to cancer cell surface receptors and exerting a good inhibitory effect on LC cells (114). Compared to the neutral components RBP-1, the acidic polysaccharide component RBP-2, RBP-3 extracted from *Radix Bupleuri* shows stronger macrophage activation ability, which is closely related to its ability to bind TLR2/4 and activate downstream MAPK and NF-κB signaling pathways (12). Recent structural analysis of arabinogalactan protein-pectin complexes from pine (Pinus sylvestris) revealed that the presence of galacturonic acid and rhamnogalacturonan-I domains is critical for maintaining the polysaccharide conformation and bioactivity, which may also contribute to their immunomodulatory and anti-cancer properties (108).

The complexity of the monosaccharide composition directly affects the selectivity of the anti-cancer mechanism. For example, the red apple polysaccharide RRTP80–1 composed of arabinose, glucose, and galactose activates the immune system, upregulates ROS and NO to inhibit tumor angiogenesis; while β-glucan, which contains only glucose, directly induces cancer cell apoptosis (109). Fungal polysaccharides are rich in β-(1→3)-D-glucan, which is a classic biological response modulator, activating innate immune cells by recognizing Dectin-1 receptor and inducing cancer cell apoptosis (110). Minor composition differences can lead to changes in the anti-cancer spectrum, such as the specific molar ratio (arabinose: galactose: xylose: glucose: mannose) of *Rubus chingii Hu* polysaccharide R1, which enables it to have both direct killing and immune regulation dual effects (180). Marine polysaccharides such as carrageenan (dominated by fucosyl groups and containing arabinose, galactose, glucose, xylose, mannose, and glucuronic acid) regulate apoptosis through multiple signaling pathways, inhibit metastasis, and enhance chemotherapy efficacy (181). In summary, the monosaccharide composition not only affects the physicochemical properties of polysaccharides but also determines their specific recognition with immune receptors, thereby regulating anti-cancer activity.

### 5.2 Molecular weight

The anticancer activity of polysaccharides is not linearly correlated with MW but rather exhibits an optimal MW range. The MW in the range of 10–50 kDa often confers the strongest activity. For instance, LBP-3 (40 kDa) achieved a significantly higher *in vivo* inhibition rate against H22 hepatoma cells (37.97%) compared to lower or higher MW fractions (9.09%-18.18%) (182). This optimal range likely represents a balance between maintaining an active conformation and enabling efficient transmembrane transport. In contrast, low-MW polysaccharides (<50 kDa) tend to act directly on cancer cells. Their shorter chains and higher branching facilitate penetration of the tumor barrier and direct entry into cells to trigger apoptosis. For example, NIPGF01 from *Grifola frondosa* (48.6 kDa) induces apoptosis in GC cells (MGC80-3) at a rate of 79.2% at 800 μg/mL (110).

Conversely, high-MW polysaccharides (>200 kDa) typically exert indirect, immunomodulatory effects. Their longer chains favor the formation of ordered structures (e.g., triple helices), enhancing recognition by immune cells and immune surveillance. *Ginseng*-derived PGPW1 (350 kDa) inhibits T24 bladder cancer cell metastasis by downregulating the M3 muscarinic receptor (183). High-MW laminarin from brown algae suppresses colony formation in SK-MEL-28 melanoma and DLD-1 colon cancer cells by modulating MMP-9 and ERK/MAPK signaling (184). Similarly, high-MW hyaluronic acid binds CD44^+^ receptors to inhibit glioblastoma (185). However, excessively high MW (>1000 kDa) increases viscosity and reduces solubility, compromising bioactivity. Thus, precise MW control is key to optimizing the anticancer potential of polysaccharides.

### 5.3 Type and connection mode of glycosidic linkages

The configuration (α/β), linkage positions, and branching patterns of glycosidic bonds are core structural determinants of the anticancer activity of polysaccharides, governing chain conformation, receptor recognition, and bioactivity (186). The β-(1→3)-D-glucan backbone with β-(1→6)-linked branches represents a classic bioactive motif. For instance, lentinan-featuring a β-(1→3)-glucan main chain with two β-(1→6)-branches per every five glucose residues-adopts a triple-helix conformation essential for its anti-tumor activity (187); disruption of this helix abolishes activity without altering the primary structure (115). Similarly, schizophyllan (from *Schizophyllum commune*) relies on this structural arrangement for conformational stability (187).

In contrast, heteropolysaccharides containing (1→4)- and (1→6)-linkages exhibit distinct mechanisms. LS-P from *Lepista sordida*, rich in (1→4)- and (1→6)-glycosidic bonds, promotes lymphocyte and macrophage proliferation and disrupts the cytoskeleton of gastric cancer cells (116). TS-P from *Trametes sanguinea*, characterized by a (1→4)-α-D-glucose/(1→4,6)-β-D-glucose backbone, shows enhanced immunostimulatory activity attributed to its high (1→4)-linkage content (9). Notably, grifolan (from *Grifola frondosa*) possesses an inverted structure-a β-(1→6)-linked main chain with β-(1→3)-branche, despite lower potency than lentinan, retains significant anti-tumor activity due to its high molecular weight and triple-helix conformation (117). α-Glycosidic linkages also contribute to anticancer activity, often requiring structural synergy or chemical modification. YCP, a polysaccharide from the marine fungus *Phoma herbarum*, features an α-(1→4)-linked backbone with α-(1→6)-branches and activates B-cells via TLR2/4; intriguingly, its degradation products exhibit receptor selectivity switching to TLR4 dependence (113). CP2-S from *Cordyceps militaris* shares this α-(1→4)-glucan structure and displays immunostimulatory properties (188). However, ACPA1 from *Actinidia chinensis* roots, despite containing α-(1→4) and α-(1→6) linkages, shows weak immunostimulatory activity unless potentiated by sulfation, underscoring the role of chemical modification in compensating for inherent structural limitations (113).

Beyond these, glycosidic linkage patterns critically influence the bioactivity of pectins and marine polysaccharides. In pectins, the RG-I domain, comprising an alternating α-(1→4)-galacturonic acid and α-(1→2)-rhamnose backbone with arabinose/galactose side chains, confers anticancer properties (108). Fucoidans, with (1→3)- and (1→4)-linked L-fucose backbones and galactose branches, exhibit antioxidant and antiviral activities (189).

### 5.4 Spatial conformation

The spatial conformation of polysaccharides represents an advanced structural determinant of their anticancer activity, with the triple-helix conformation garnering particular interest for its ability to precisely recognize pattern recognition receptors on immune cells (190). WAAP-2, isolated from *Agaricus bisporus*, exhibits a typical triple-helix structure. It significantly induces apoptosis in HT-29 colon cancer cells by upregulating Caspase-3 and Bax while downregulating Bcl-2, and it inhibits migration and invasion (190). Similarly, AHP-3a, an acidic polysaccharide from *Alpinia officinarum*, also adopts a triple-helix conformation, efficiently suppressing proliferation and metastasis of HepG2 and Huh-7 hepatoma cells without cytotoxicity to normal cells (114). TFP, derived from *Tremella fuciformis*, possesses a triple-helix structure with a molecular weight of approximately 13 kDa, defined by specific glycosidic linkages in its main chain. This conformation not only confers antioxidant activity but also drives macrophage M1 polarization via the MAPK and NF-κB pathways, enhancing phagocytosis and inhibiting tumor growth in a melanoma-macrophage co-culture system (191).

Beyond the triple helix, other conformations also contribute uniquely to anticancer efficacy. Although the single-helix conformation is comparatively less stable, it retains significant activity under certain conditions (192, 193). For instance, linear (1→3)-β-D-glucan from *Auricularia auricula* exists as a single helix and exhibits potent anti-tumor effects. The random coil conformation, commonly found in low-MW or denatured polysaccharides, offers distinct advantages (192). Degraded fucosylated chondroitin sulfate, for example, adopts an extended conformation with exposed active sites, facilitating cellular uptake and enhancing inhibition of A549 lung cancer cells (194).

These findings collectively demonstrate that spatial conformations, ranging from ordered triple-helices to relatively flexible random coils, underpin the anticancer activity of polysaccharides by modulating receptor recognition, signal pathway activation, and cellular uptake efficiency.

### 5.5 Chemical modification

Chemical modification by introducing functional groups such as sulfate, acetyl, carboxymethyl, phosphate, or selenium can significantly enhance the anticancer activity of natural polysaccharides (111, 195).

Sulfation, the most extensively studied approach, introduces sulfate groups (-SO_4_²^-^) to enhance anti-tumor activity (196). For instance, sulfated polysaccharides from *A.* sphaerocephala block the cell cycle, inhibit adhesion and migration, and modulate MAPK and NF-κB signaling (197). The degree of substitution is critical: moderate sulfation optimizes electrostatic interactions with receptors, whereas excessive sulfation may cause chain degradation or toxicity (198). Acetylation introduces acetyl groups (-COCH_3_), altering the hydrophobic microenvironment and hydrogen-bonding network. Acetylated polysaccharides from *Cyclocarya paliurus* regulate cytokine secretion, enhance immune surveillance, and suppress tumor growth (198). This modification also shows therapeutic potential in aging, infection, and other pathological conditions (199). Carboxymethylation introduces carboxymethyl groups (-CH_2_COOH), improving water solubility and bioactivity (200). Carboxymethylated *Polyporus umbellatus* polysaccharide exerts anti-tumor effects via NF-κB, Nrf2-ARE, and MAPK/P38/JNK pathways (201). Carboxymethylated *Ganoderma lucidum* polysaccharide exhibits an IC_50_ of 38 μg/mL against S-180 sarcoma cells and chelates transition metal ions, thereby impairing tumor oxidative stress repair (202).

Phosphorylation introduces phosphate groups (-PO_4_³^-^), enhancing solubility, antiviral activity, and antioxidant capacity. Phosphorylated polysaccharides from *Codonopsis pilosula*, comprising (1→3)-β-D-glycosyl, (1→2,3)-β-D-glycosyl, and (1→3)-α-D-rhamnosyl residues, exhibit dual antiviral and anti-tumor activities (200). Selenization is an emerging strategy that incorporates selenium into polysaccharide backbones, forming selenopolysaccharides (203). Selenium nanoparticles (SeNPs) conjugated with polysaccharides induce mitochondrial apoptosis in various cancer cells (e.g., HepG2, MCF-7) by upregulating pro-apoptotic proteins (Bax, caspase-3), downregulating anti-apoptotic Bcl-2, and disrupting mitochondrial membrane potential (204). For example, laminarin-modified SeNPs show an IC_50_ of 23.4 ± 2.7 μM against HepG2 cells, significantly outperforming unmodified SeNPs (203). Combined modifications and nanoformulations further expand therapeutic applications. Co-application of carboxymethylation and sulfation can produce synergistic effects (198), while folate-conjugated selenium nanoparticles achieve targeted, low-toxicity anti-tumor efficacy by binding to folate receptors on cancer cells (205). These strategies collectively advance the clinical potential of polysaccharide-based therapeutics.

In conclusion, the anti-cancer activity of polysaccharides arises from the integrated and synergistic effects of multiple structural determinants, including monosaccharide composition, molecular weight, glycosidic linkages, spatial conformation, and chemical modification. A comprehensive understanding of these structures, activity relationships is indispensable for the rational design of polysaccharide-based antitumor agents that exhibit high potency and low systemic toxicity. Moreover, such mechanistic insight establishes a robust conceptual and strategic foundation for the targeted engineering and site-selective chemical modification of bioactive polysaccharide derivatives, thereby accelerating their translation from bench to bedside in tumor prevention and adjunctive cancer therapy.

## 6 Clinical application of polysaccharide-based drugs

(**Table 4**) Polysaccharide drugs have entered clinical practice and trials, leveraging their core mechanisms-immune cell regulation, apoptosis induction, and proliferation inhibition. Several products are already marketed, including *Ganoderma lucidum* polysaccharide tablets, *Grifola frondosa* capsules, and Poria cocos polysaccharide oral liquid, demonstrating promising applications.

**Table 4:** Clinical exploration of polysaccharides in the treatment of cancer.

Polysaccharides	Cancer	Clinical stage	Administration way	Dosage form	Ref
BG136	Advanced solid tumors	Phase I (completed), Phase II (completed)	Injection	Injectable solution	(206)
Lentinan	GC, CRC, various solid tumors	Marketed	Injection, Oral	Injectable solution, Tablet	(207)
APS (PG2)	Gynecological tumors, BC, cancer-related fatigue	Marketed (approved)	Injection	Injectable solution	(209)
PSK (Krestin)	GC, CRC, NSCLC	Marketed (Japan)	Oral	Oral formulation	(207)
Chitosan-based dressings	Skin wounds (cancer surgery wounds)	Marketed	Topical	Liquid dressing	(210–213)
Sodium alginate sulfate ester (Drug 911)	AIDS, Hepatitis B	Clinical exploration	Injection/Oral	Injectable solution/Oral liquid	(215)
Yeast β-glucan + Erbitux	Metastatic CRC	Clinical trial (ongoing)	Injection	Combination therapy with monoclonal antibody	(211, 216)
Maitake D-Fraction	BC (triple-negative)	Preclinical/Early clinical	Oral/Injection	Oral supplement/Injectable	(216)
Sodium alginate sulfate propylene glycol	Various indications (anti-coagulant, anti-viral)	Marketed	Oral/Injection	Modified polysaccharide drug	(217)
Ganopoly (*Ganoderma lucidum* polysaccharide)	Type 2 diabetes (metabolic disease)	Clinical trial completed (2004)	Oral	Capsule/Tablet (1800 mg/day, three divided doses)	(218)
*Ganoderma lucidum* polysaccharide tablets	Various cancers (adjunctive therapy)	Marketed	Oral	Tablet	–
*Grifola frondosa* capsules	Various cancers (immunomodulation)	Marketed	Oral	Capsule	–
*Poria cocos* polysaccharide oral liquid	Various cancers (adjunctive therapy)	Marketed	Oral	Oral liquid	–

In innovative drug development, BG136 for injection-the world’s first marine-derived immunomodulatory anti-tumor polysaccharide-has completed preclinical research and received clinical approval as a Class I drug. Phase I trials in advanced solid tumors showed good safety with an objective response rate of 18%. Phase II trials are complete, and it is expected to provide a new treatment option for cancer patients (206). Several mushroom-derived polysaccharides have been developed into clinical drugs with distinct immunomodulatory and anti-cancer applications. Lentinan, a β-glucan from *Lentinus edodes*, is widely used in injectable and tablet forms. It activates the TLR4/NF-κB pathway to promote IFN-γ and IL-2 release, thereby enhancing immunity, reducing chemotherapy side effects, and lowering drug resistance (207). APS (trade name PG2) is another important variety. Having completed clinical trials, it has been approved for marketing, demonstrating unique value in alleviating cancer-related fatigue and regulating immunity. Clinical studies indicate that PG2 is safe and well-tolerated in patients with gynecological tumors during chemotherapy. Specifically, in pre-menopausal breast cancer patients, it significantly improves chemotherapy-induced fatigue and insomnia, enhancing overall health status and quality of life (209).

Protein-bound polysaccharide K (PSK, trade name Krestin), a protein-polysaccharide complex extracted from *Coriolus versicolor*, is widely used in Japan as an adjunctive treatment for GC and CRC. Clinical studies and meta-analyses confirm that PSK combined with postoperative adjuvant chemotherapy prolongs patient survival. In NSCLC, PSK extends the remission period when combined with chemotherapy. Additionally, it has proven effective against various other cancers by reducing chemotherapy side effects and improving quality of life (207).

Polysaccharide materials also show progress in medical devices: chitosan-based liquid dressings have achieved technology transfer and market launch, significantly increasing skin wound healing rates in clinical settings (210, 212, 213). Lentinan, a mature polysaccharide, is widely used clinically in injection and tablet forms. Studies confirm that lentinan activates the TLR4/NF-κB pathway, promoting IFN-γ and IL-2 release (214), enhancing immunity, alleviating side effects, and reducing drug resistance during adjuvant chemotherapy. APS, another important variety, has completed clinical trials and been approved for marketing, demonstrating unique value in alleviating cancer-related fatigue and immune regulation. Clinical studies show that gynecological cancer patients undergoing chemotherapy tolerate APS safely (209).

Sodium alginate derivatives exhibit diverse functions in clinical applications. For instance, sodium alginate sulfate ester (Drug 911), explored in China for anti-AIDS therapy, inhibits viral reverse transcriptase and prevents viral adsorption by interacting with the viral surface glycoprotein; additionally, it demonstrates potential against hepatitis B by inhibiting viral DNA polymerase (215). In another case, yeast β-glucan is under clinical trial for metastatic colorectal cancer when combined with anti-tumor monoclonal antibodies (e.g., Erbitux). It enhances neutrophil-mediated tumor cell killing by activating complement receptor 3 (211). Furthermore, the Maitake D-Fraction has shown significant anti-tumor and anti-metastatic activity in preclinical breast cancer studies, where it upregulates E-cadherin expression and promotes β-catenin membrane localization to inhibit the invasion and metastasis of triple-negative breast cancer cells (216).

Despite good biocompatibility, polysaccharides often suffer from low water solubility and stability, limiting their biological activity. Chemical modification can improve these properties. For instance, the marketed sodium alginate sulfate propylene glycol significantly enhances its biological activity by introducing sulfonic acid groups and propylene glycol ester groups into the sodium alginate oligomer molecules (217). A clinical trial completed in 2004 evaluated Ganopoly, a polysaccharide extract from *Ganoderma lucidum*, in 71 patients with type 2 diabetes. Administered orally at 1800 mg per day (in three divided doses) for 12 weeks, the treatment significantly reduced glycated hemoglobin (HbA1C) and blood sugar levels. This demonstrates the potential of polysaccharide-based drugs in the clinical management of metabolic diseases (218, 219).

Overall, although polysaccharide drugs demonstrate significant anti-cancer potential through multi-target mechanisms, the transition from the laboratory to clinical application still faces challenges: low bioavailability, unclear structure-activity relationships, insufficient understanding of the mechanisms, and the lack of long-term safety data. Future research should utilize advanced formulation technologies (such as liposomes, polymer micelles) to enhance stability and targeting properties, and clarifying the molecular mechanisms will provide a more solid theoretical foundation for clinical application. Although various polysaccharide preparations (such as Lentinan, APS, PSK, and BG136) have entered clinical use and shown good safety and immunomodulatory properties, the clinical transformation of new polysaccharide anti-cancer drugs is still limited. The key challenges include low bioavailability, unclear structure-activity relationships, and insufficient understanding of the mechanisms. Future work should prioritize standardized extraction and modification protocols, advanced formulation technologies (such as nanoparticle delivery systems), and strong clinical evidence to establish long-term efficacy and safety.

## 7 Conclusions and perspectives

Due to their excellent biological safety and low toxicity, polysaccharide-based drugs have become promising candidate for anti-tumor treatment and adjuvant therapy. Extensive research has confirmed their significant therapeutic effects on a range of common malignancies, including LC, GC, LC, OC, and CRC.

Despite these hopes, clinical translation faces several challenges. Their structural complexity, including molecular chain conformation and modification patterns, greatly affects biological activity and complicates mechanistic elucidation. Current clinical trials often have small sample sizes and lack long-term efficacy and safety data. The potential risks associated with long-term administration need to be further verified. Future research should emphasize in-depth mechanistic studies and high-quality clinical evidence to accelerate clinical translation.

Among these, chemically modified polysaccharides (such as sulfated, phosphorylated, etc.), low-molecular-weight derivatives and acidic polysaccharide fractions demonstrate optimal anti-cancer activity, benefiting from their enhanced bioavailability and specific target-binding capacity. Polysaccharides exert anti-tumor effects through synergistic mechanisms: immunomodulation (e.g., M1 macrophage polarization, T cell activation), induction of tumor cell cycle arrest and apoptosis, inhibition of migration/invasion and angiogenesis, as well as regulation of key signaling pathways including P53, NF-κB and Wnt/β-catenin, etc. These multi-target and low-toxicity characteristics solidify their potential as promising anti-cancer candidates.

This review systematically summarizes the anti-tumor mechanisms, core signaling pathways and selective cytotoxicity of polysaccharides from multiple sources, and further expounds their clinical translation progress and application examples, which constructs a comprehensive theoretical framework for the development of polysaccharide anticancer preparations and provides clear research directions for subsequent in-depth exploration of polysaccharide anti-tumor efficacy. A major challenge is that the structural complexity of polysaccharides means their mechanisms of action are not yet fully elucidated, and the current clinical research of polysaccharide anticancer drugs is limited by small sample size and lack of long-term safety data. The clinical evidence remains limited to small-scale trials with insufficient long-term safety data. These gaps underscore the need for more rigorous, structure-defined investigations and well-designed clinical studies to advance the field. In addition, the poor water solubility and stability of partial polysaccharides also restrict their clinical transformation and industrial application, issues that need to be addressed in future studies through structural modification and the development of novel delivery systems.

To address the existing limitations of polysaccharide-based anticancer research, future studies should concentrate on several key directions. First, advanced analytical techniques, glycomics, AI-assisted modeling, homogeneous fractions, multi-omics and genetic tools should be employed to decipher the structure-activity relationship and clarify the precise molecular mechanisms. Second, large-sample, long-term, multi-center Phase II/III clinical trials with extended follow-up are needed to supplement systematic safety and efficacy data. Meanwhile, unified quality control standards should be established, and novel delivery systems and chemical modifications developed to enhance bioavailability and stability. These targeted efforts will provide clear technical routes and solid foundations for the rational development and clinical translation of polysaccharide-based anticancer drugs.
